# Neuroblastoma Heterogeneity, Plasticity, and Emerging Therapies

**DOI:** 10.1007/s11912-022-01270-8

**Published:** 2022-04-01

**Authors:** Kristina Ihrmark Lundberg, Diana Treis, John Inge Johnsen

**Affiliations:** Childhood Cancer Research Unit, Department of Women’s and Children’s Health, Karolinska Institutet, 17177 Stockholm, Sweden

**Keywords:** Tumor heterogeneity, Tumor plasticity, Tumor microenvironment, Differentiation therapy, ALK inhibitors

## Abstract

**Purpose of Review:**

The evolving information of the initiation, tumor cell heterogeneity, and plasticity of childhood neuroblastoma has opened up new perspectives for developing therapies based on detailed knowledge of the disease.

**Recent Findings:**

The cellular origin of neuroblastoma has begun to unravel and there have been several reports on tumor cell heterogeneity based on transcriptional core regulatory circuitries that have given us important information on the biology of neuroblastoma as a developmental disease. This together with new insight of the tumor microenvironment which acts as a support for neuroblastoma growth has given us the prospect for designing better treatment approaches for patients with high-risk neuroblastoma. Here, we discuss these new discoveries and highlight some emerging therapeutic options.

**Summary:**

Neuroblastoma is a disease with multiple facets. Detailed biological and molecular knowledge on neuroblastoma initiation, heterogeneity, and the communications between cells in the tumor microenvironment holds promise for better therapies.

## Introduction

Neuroblastoma is the most common and deadly tumor of infancy accounting for 15% of all childhood cancer-related deaths [1]. Neuroblastomas almost exclusively occur in young children and the median age for diagnosis is 18 months. Approximately 40% of the patients are younger than 1 year at diagnosis whereas less than 5% are older than 10 years [2, 3]. Neuroblastoma is characterized histopathologically as a “small round blue cell” tumor, an entity of neoplasms consisting mainly of pediatric solid cancers. Clinically, neuroblastoma manifests as a primary tumor anywhere along with the sympathetic nervous system, with > 50% occurring in the adrenal medulla [1]. Neuroblastomas are heterogeneous diseases, which also is reflected in patient survival. Low-intermediate risk patients have an overall survival rate of > 95%, whereas high-risk patients have < 50% long-term survival [4].

The clinical heterogeneity observed in neuroblastoma can be reflected through chromosomal aberrations. Low-risk patients commonly present with whole chromosomal gains and the tumor cells are frequently hyperdiploid, whereas high-risk patients have a chromosomal makeup consisting of segmental chromosomal gains or losses [5–9]. The most frequent chromosomal aberrations associated with high-risk patients and poor prognosis in neuroblastoma are segmental gain of chromosome 17q, hemizygous loss of chromosome 1p and 11q, and somatically acquired amplification of the oncogene *MYCN* [3]. Additionally, high-risk neuroblastoma can also present with rearrangements at chromosomal region 5p15.33 that is located close to the telomerase reverse transcriptase gene (*TERT*) [10, 11]. Similar to the majority of childhood cancers, neuroblastomas show low somatic mutation counts (12–18, median 15) and there is no single mutation that acts as a driver for the development of all neuroblastomas [12–14]. The *ALK* (anaplastic lymphoma kinase) gene harbors the most frequently detected somatic mutations in neuroblastoma, found in 8–10% of neuroblastoma cases. Mutations of *ALK* are also present in familial neuroblastomas which encompass 1–2% of the neuroblastoma patients [15]. Additionally, germline loss-of-function mutations of the paired-like homeobox 2B (*PHOX2B*) gene have been found in familial neuroblastoma as well as in approximately 4% of spontaneous neuroblastomas [12, 14, 15]. Recurrent genetic alterations have also been observed for *LIN28B*, *ATRX*, *ARID1A/1B*, *BARD1*, *LMO1*, and *TP53* [12–14, 16–18] (Table 1).

**Table 1:** Recurrent gene amplifications and mutations in neuroblastoma

Genetic alterations	Gene	Chr. location	Additional information
Amplification	*MYCN*	2p24	HR, oncogenic driver (⁓ 20%)
Amplification	*ALK*	2p23	HR (⁓ 4%)
Polymorphism	*LIN28B*	6q16	HR
Rearrangements	*TERT*	5p15.33	Promoter rearrangements-enhancer hijacking, HR (⁓ 25%)
Loss-of-function mutations/deletions	*ATRX*	Xq21.1	(⁓ 10%), frequent in older HR patients
Activating mutations	*ALK*	2p23.2-p23.1	7–10% somatic mutations, 1–2% germline mutations, HR
Loss-of-function mutations	*PHOX2B*	4p13	Both germline and somatic mutations
Mutations/LOH	*ARID1A*	1p35.3	(7%), associated with poor survival
Mutations/Deletions	*ARID1B*	6q25.1	(6%), associated with poor survival
Polymorphism	*BARD1*	2q35	HR patients
Mutations/duplications	*LMO1*	11p15.4	(12%), HR patients
Inactivating mutations	*TP53*	17p13.1	1–2% mutations in primary tumors, 10% in recurrent/relapsed tumors

Data collected from ref [12–18]. Abbrev: *LOH*, loss of heterozygosity, *Chr*.; chromosomal, *HR*; high risk

Further adding to the complexity of the disease is the presence of frequent inter- and intra-tumorigenic heterogeneity in individual patients and the accumulation of gene mutations observed in recurrent and relapsed tumor tissues [17–20]. The heterogeneity observed in neuroblastoma is a clinical challenge since tumors that are phenotypically and morphologically alike may respond fundamentally differently to therapy, depending on their molecular makeup. This proposes that neuroblastoma patients in general and specifically those diagnosed with high-risk disease should be carefully examined with regard to their molecular landscape in order to design individual therapies targeting the observed molecular aberrations in addition to current conventional therapies [2]. Here we discuss some recent developments in the understanding of neuroblastoma initiation, heterogeneity, plasticity, and communication between cells in the tumor supporting microenvironment that will have an impact when designing new therapies.

## The Neural Crest and Neuroblastoma Cell of Origin

Neuroblastoma is an embryonic cancer originating from cells in the neural crest, a transient structure that appears at the margins of the closing neural tube, consisting of multipotent stem cells active during early embryonic development. Neural crest cells give rise to a number of different cell types including neurons and glial cells within the peripheral nervous system, mesenchymal, pigment and secretory cells, and bone and cartilage cells of the face [21]. During embryonal development, neural crest cells need to undergo epithelial-to-mesenchymal transition and migrate extensively from the neuroepithelium to more distant locations in the embryo where the cells finally maturate and differentiate into established structural and functional networks [22]. The fact that the neural crest is a transient structure has complicated the search for the neuroblastoma cell of origin.

The combination of timing of the onset of disease and its clinical presentation brought about the consensus that neuroblastoma most likely originates from symphatoadrenal progenitor cells within the neural crest that normally differentiate to sympathetic ganglion cells and adrenal catecholamine-secreting cells [23]. The adrenal gland medulla contains secretory cells named chromaffin cells that synthesize and store hormones, including catecholamines [24]. Morphologically, these cells lack neurites and resemble endocrine cells. Earlier studies established the consensus that both adrenal chromaffin cells and sympathetic neurons originate from sympathoadrenal progenitor cells [25].

**Fig. 1:**
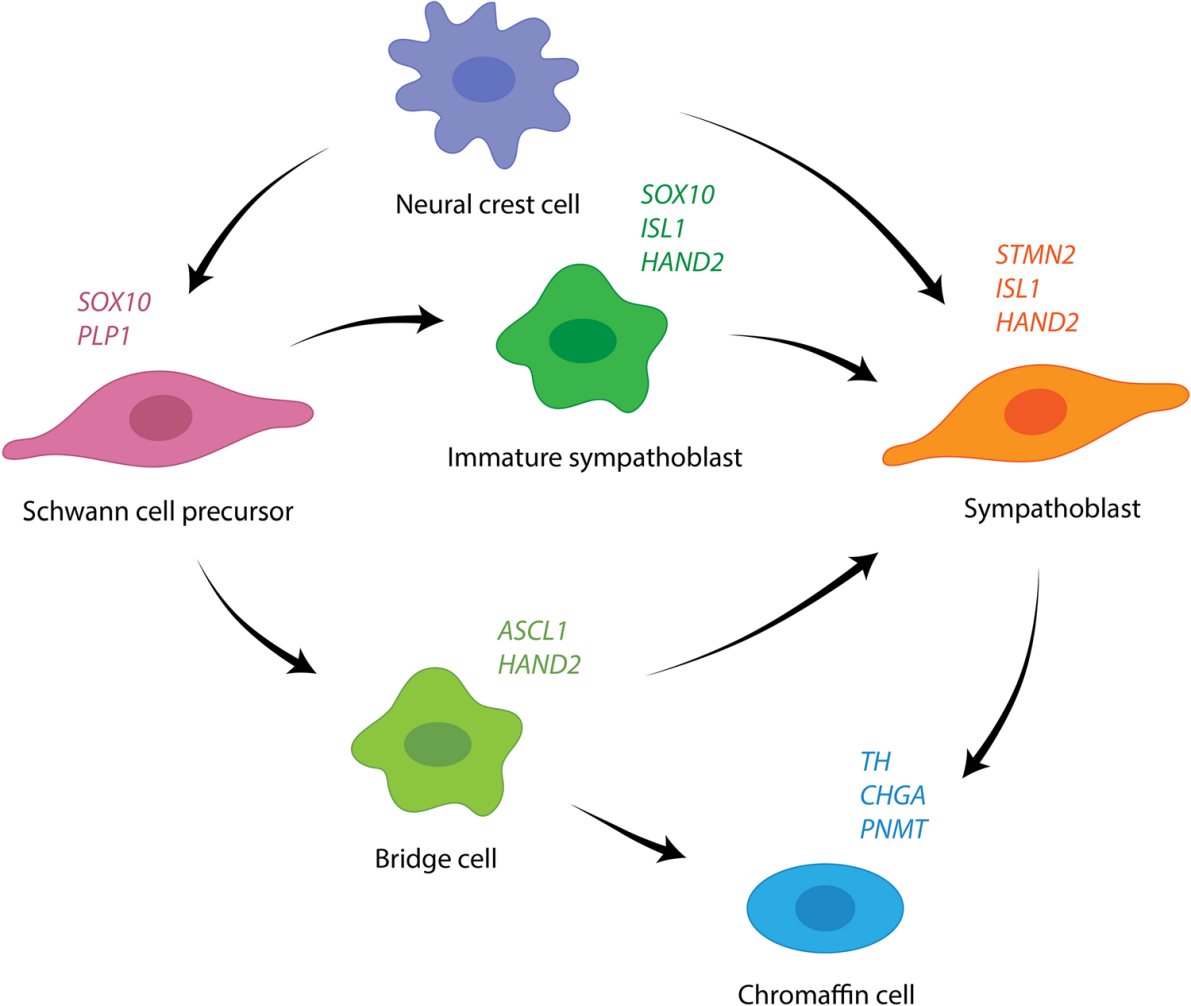
Normal development of the sympathoadrenal lineage in humans. During neural crest development, Schwann cell precursor (SCPs) differentiate into both chromaffin cells and sympathoblasts [27•, 29•]. The cell annotations in capital letters are genes that were expressed by the corresponding cells. SCPs, sympathoblasts, and chromaffin cells expressed these genes in both studies

However, this was recently challenged by data demonstrating that Schwann cell precursors deriving from migrating neural crest cells are the ancestors of adrenal medullar chromaffin cells and fetal adrenal neuroblasts (Fig. 1) [26, 27•]. Recent data derived from single-cell RNA sequencing analyses comparing malignant neuroblastoma with neural crest cell signatures resulted in the identification of two different immature cell populations within the neural crest as potential cells of origin for neuroblastoma [28•, 29•, 30•]. One study indicated that neuroblastoma has a predominant chromaffin-cell-like phenotype and that these cells are the cell of origin [28•], whereas two other studies suggested that neuroblastomas transcriptionally resemble normal fetal adrenal neuroblasts (also named sympathoblasts) [29•, 30•].

Historically, Schwann cell precursors were considered to be primed toward Schwann cell differentiation. However, recent studies suggest that these precursors may give rise to several different cell types including melanocytes, odontoblasts, parasympathetic and enteric neurons, and endoneural fibroblasts in addition to chromaffin cells and neuroblasts (sympathoblasts) [27•, 31–35]. These studies suggest that the plasticity of Schwann cell precursors is more extensively involved in tissue establishment and regeneration than previously assumed [25]. Although these findings have increased our understanding of human sympathoadrenal development and given further insight into the heterogeneity of neuroblastoma, the trajectory from developing normal neural crest cells to malignant neuroblastoma is still incomplete [36].

## Neuroblastoma Heterogeneity and Plasticity

Neuroblastomas are characterized by a high degree of heterogeneity which is reflected in clinical presentations spanning from spontaneous regression or differentiation to treatment-refractory progression regardless of intensive multimodal therapies. The spontaneous regression of tumor cells observed in a subgroup, named 4S, of neuroblastoma patients is exceptional among human cancers. Stage 4S neuroblastomas are found in young children up to 18 months of age and usually present with small abdominal tumors with several metastases to the liver, skin, and bone marrow [37]. The majority of stage 4S patients undergo spontaneous regression of their tumor cells with limited or no treatment. The mechanisms for this spontaneous regression are not fully known but epigenetic regulation, neurotrophin deprivation, loss of telomerase activity, or immune responses have been proposed factors for induction of regression [38]. Of note, the spontaneous regression seen in stage 4S neuroblastomas resembles events observed within the neural crest where excess precursor cells undergo apoptosis during the final stages of maturation [39].

Although spontaneous regression occurs in some patients, the majority of neuroblastomas are diagnosed as high risk and most of these patients have metastatic disease already at diagnosis suggesting that induction of metastasis is an early event [3].

Although neuroblastomas have a low mutational burden compared to most adult cancers, recent data have given evidence for the presence of intra-tumor heterogeneity and different evolutionary trajectories with discrete genotypes in different locations of the same tumor [40•]. Also, a comparison of treatment-naïve primary neuroblastomas with matched relapsed tumors demonstrated increased mutational burden at relapse including genes within the RAS-MAPK signaling pathway, Hippo-Yap pathway, and epithelial-mesenchymal transition processes that were not present at initial diagnosis [17, 18]. Adding to this complexity is the recent demonstration of at least two distinct tumor cell types deriving from different cell lineages, adrenergic (ADRN) and mesenchymal (MES) which differ in transcriptomic, phenotypic, and super-enhancer expression [41, 42]. Similarly, a more recent study detected the same cell types in primary neuroblastomas but also discerned additional subtypes in the ADRN group [43•]. The ADRN tumor cells could be further divided into *MYCN*-amplified, *MYCN* non-amplified high-risk, and *MYCN* non-amplified low-risk. These subtypes correlated to different clinical outcomes, whereas the *MYCN*-amplified subtype correlated with the worst outcome.

MES cells have been shown to resemble neural crest cell precursors, have an active NOTCH signaling pathway, and seem to be more resistant to chemotherapy [41]. They also occur more frequently in relapsed tumors and can be induced in vitro by activation of RAS [43•]. In *MYCN*-amplified cell lines, six members of the transcriptional core regulatory circuitry that are controlled by super-enhancers have been identified: *HAND2*, *ISL1*, *PHOX2B*, *GATA3*, *ASCL1*, and *TBX2* [41, 42, 44–46]. *MYCN* regulates the expression of all these genes and acts as a general amplifier of the transcriptional circuitry. These six-core regulatory circuitry transcription factors are expressed in both ADRN and MES cells, although somewhat less in the MES subtype [44]. ADRN and MES cells have been shown to bi-directionally interconvert between the different cell states in culture, suggesting plasticity of neuroblastoma cells. This process, reminiscent of epithelial-to-mesenchymal transition, has been named noradrenergic-to-mesenchymal transition and probably relies on epigenetic reprogramming [47].

All these studies on intra-tumor heterogeneity and different neuroblastoma cell states have focused on the neoplastic cells, whereas the biological and molecular landscape of the non-tumorigenic cells residing in the tumor microenvironment and their molecular communication with the tumor cells has not been investigated until recently. The importance of the non-tumorigenic cellular niche can be illustrated by the demonstration that neuroblastoma cells from the same batch exhibit highly dissimilar growth depending on where they grow. Subcutaneous injection of neuroblastoma cells gives rise to non-invasive cell expansions, whereas identical cells injected orthotopically result in tumor cell expansion with metastatic spread [48]. Similarly, implanting of tumor cells deriving from one single neuroblastoma sample as patient-derived orthotopic xenografts resulted in differences in transcriptional profiles and divergent tumor growth despite the fact that the injected cells should be genetically identical and contain a similar population of ADRN and MES cells [19]. These data point to the assumption that the composition of stromal and immune cells present in the tumor microenvironment has important functions in regulating the tumor cell behavior, cell-state composition, and possibly intra-tumor heterogeneity.

## The Tumor Microenvironment in Neuroblastoma

The progress in designing more precise targeted therapies including the striking developments in immunotherapies for certain cancers has spawned the focus on the nature and function of the tumor microenvironment (TME). The paucity of recurrent mutations and oncogene activation in pediatric cancers, including neuroblastoma, poses a major challenge for developing personalized medicine and also limits the effects of checkpoint inhibitors as a treatment option for these cancers. Moreover, since neuroblastoma is an embryonic tumor, an immature or impaired immune system can further complicate the design of successful immunotherapies [49•]. Given the enhanced recent focus on immunotherapy, the innate and adaptive immune cells including T and B lymphocytes, tumor-associated macrophages, dendritic cells, natural killer cells, and natural killer T cells have been given much attention when studying cells within the microenvironment of neuroblastoma. However, non-immune cells like endothelial cells, cancer-associated fibroblasts, pericytes, and mesenchymal stem cells have also recently been shown to have important functions in tumor progression and induction of therapy resistance [50]. Moreover, alterations of the extracellular matrix (ECM) have been reported to be mediators of tumor progression in neuroblastoma by increased collagen cross-linking influencing morphological changes [51]. Also, recent data suggest that extracellular vesicles released by cells in the tumor microenvironment carry oncogenic microRNAs as well as proteins involved in tumor progression such as CD147, a transmembrane protein involved in metastasis, and CD276/B7-H3, an immune checkpoint protein enabling neuroblastoma cells to evade NK cells [50, 52, 53].

The different technological approaches used to study the immune cell landscape in neuroblastoma have sometimes generated conflicting data, and therefore, the pros and cons of the different populations of immune cells detected in neuroblastoma tissues have been difficult to interpret. Nevertheless, a number of studies in neuroblastoma have shown that the immune system plays a critical role in both prognosis and response to treatment.

## MYCN Influences the Immune Microenvironment in Neuroblastoma

A general picture of the tumor microenvironment in neuroblastoma is the differences in the cellular landscape of *MYCN*-amplified vs *MYCN* non-amplified neuroblastomas [50]. MYCN has been shown to inhibit the expression of MHC class I antigens that contribute to tumor antigen presentation necessary for immune cells to recognize and attack tumor cells [54]. This lack of antigen presentation in *MYCN*-amplified neuroblastomas is responsible for the escape from cytotoxic T cell and interferon-mediated immune responses [55]. *MYCN*-amplified neuroblastomas have also been reported to contain less CD4 + and CD8 + T cells, dendritic cells, NK cells, and macrophages compared to non-*MYCN*-amplified neuroblastomas [56, 57]. Also, T cell receptor analysis showed that *MYCN*-amplified neuroblastomas had decreased levels of infiltrating lymphocyte signatures compared to non-*MYCN*-amplified tumors [58]. Furthermore, in metastatic non-*MYCN*-amplified tumors, a high level of M2 macrophages was present suggesting an increase towards M2 polarization in absence of *MYCN* expression [59]. Hence, *MYCN*-amplified tumors are poorer in immune cell infiltration compared to non-*MYCN*-amplified tumors which contain more infiltrating adaptive and innate immune cells partly through the higher expression of chemokines which are downregulated by *MYCN* [50]*.*

## Targeting the Neuroblastoma Tumor Microenvironment

Cancer-associated fibroblasts (CAFs) are characterized by expression of fibroblast activating protein (FAP), fibroblast-specific protein (FSP-1), platelet-derived growth factor receptor α and β (PDGFR α and PDGFR β), α-smooth-muscle actin (αSMA), and vimentin [60]. However, the heterogeneity in expression of these markers indicates that CAFs are polarized into distinct subpopulations depending on their origin and the local tumor environment [61, 62]. In neuroblastoma, high numbers of CAFs presented within the tumor tissue have been shown to correlate with more aggressive Schwannian stroma-poor tumors [63], and CAFs have been detected in close proximity to tumor-associated macrophages (TAMs) [63, 64]. Secretion of IL-6 from TAMs enhances the activation of STAT3 in CAFs leading to increased proliferation [64, 65]. Our laboratory has shown that prostaglandin E2 secreted by CAFs enhances the M2 polarization of TAMs and increases the proliferation of neuroblastoma cells [66–68]. Targeting microsomal prostaglandin E synthase-1 (mPGES-1), one of the important enzymes in the synthesis of prostaglandin E2, using a small molecule inhibitor, blocked the production of prostaglandin E2 from CAFs. This resulted in reduced tumor growth, inhibition of CAF migration and infiltration, and a favorable shift in the macrophage M1/M2 ratio [67]. CAFs located in the tumor microenvironment in neuroblastoma have also been reported to share functional and phenotypical characteristics with bone marrow-derived mesenchymal stem cells. These cells called CAFs-MSCs secrete several inflammatory cytokines and chemokines such as IL-6, IL-8, CCL-2, CxCL-12, and VEGF-A that promote neuroblastoma growth and enhance resistance toward chemotherapeutic drugs [64, 69].

Taken together, neuroblastomas encompass an immunosuppressive microenvironment that contains abundant CD163^+^ macrophages and C11b^+^ myeloid-derived cells [70, 71]. High-risk neuroblastoma also presents with a tumor-associated inflammatory signature that facilitates tumor growth, drug resistance, and immune evasion [59, 66, 72]. Hence, combining new modes of immunotherapies with anti-inflammatory drugs may be beneficial for this group of patients.

Except for anti-GD2 (dinutuximab) that has been included in the treatment of high-risk neuroblastoma patients for the last 20 years, no other immune-therapy approaches have proven successful so far. One important reason for this is the lack of neo-antigens present on the surface of neuroblastoma cells. An intriguing recent study may offer a solution to this. By studying the immunopeptidome in neuroblastoma, an enrichment of peptides from proteins essential for tumorigenesis was detected. This included a peptide, QYNPIRTTF, deriving from the master transcription regulator *PHOX2B*. Development of peptide-centric chimeric antigen receptors targeting this peptide resulted in selective killing of neuroblastoma cells and complete regression of neuroblastoma PDX tumors positive for PHOX2B [73••]. This promising data suggest that a peptide-centric CAR therapy can be applied on tumors with low expression of surface neo-antigens and expand the options of immunotherapeutic targets [73••].

## Differentiation of Neuroblastoma as a Treatment Option

Neuroblastoma is an embryonal tumor and can for this reason be characterized as a developmental disease where lack of differentiation or apoptosis during the development and maturation of the peripheral nervous system result in neoplastic transformation and malignant tumor growth. In fact, 13-cis retinoic acid (isotretinoin), which induces differentiation in neuroblastoma, is currently used as maintenance therapy to avoid residual disease in patients with high-risk disease. However, the effects of this treatment are limited in patients compared to the effects seen on neuroblastoma cells growing in culture. Therefore, clinical trials investigating the effects of retinoic acid in combination with other drugs or immunotherapies as well as studies to improve the retinoic acid drug delivery have been initiated [74]. The induction of tumor cell differentiation as a treatment strategy was highlighted by the recent identification of MYCN and TFAP (transcription factor AP-2) as important players in differentiation control [29•].

In *MYCN*-amplified neuroblastomas, the ADRN core regulatory circuitry of transcription factors is controlled by MYCN. This favors an immature neuroblast cell state and suppresses signals that normally induce differentiation [75]. Recent data suggest that retinoid treatment can reprogram the enhancer landscape, establishing a new retino-sympathetic core regulatory circuitry consisting of *RARA*, *HAND2*, *TBX2*, *SOX4*, *MEIS1*, and *ISL1*, causing downregulation of *MYCN* expression, proliferative arrest, and sympathetic differentiation [76]. These data provide mechanisms for the beneficial effects of retinoid treatment in neuroblastoma and explain the observed massive downregulation of *MYCN* expression seen in some neuroblastoma cells when subjected to retinoids.

During embryonal development, neural crest cells must acquire motility through the epithelial-to-mesenchymal transition. Fundamental for the motility is the Wnt-planar cell polarity (PCP) signaling cascade which guides contact inhibition of locomotion inducing polarity of cells within the neural crest. PCP proteins regulate the activity of Rho GTPases locally by stimulating or suppressing the activity of RhoA and Rac1, resulting in cells migrating away from each other upon collision [77]. Activation of Rho signaling by PCP activates rho-associated coiled-coil-containing protein kinases, ROCK1 and ROCK2 [78]. ROCK1 and ROCK2 phosphorylate downstream substrates such as myosin light chain (MLC) and LIMK1/2, which control a number of cellular functions through rearrangement of the actin cytoskeleton [79, 80]. We and others have shown that approximately 30% of neuroblastomas contain mutations in genes involved in Rho/Rac1 signaling and that high expression of ROCK2 corresponds to poor prognosis [13, 81]. Inhibition of ROCK activity induced neuroblastoma cell differentiation and suppressed cell migration, invasion, and xenograft tumor growth in mice [81]. Several ROCK inhibitors are currently undergoing clinical trials or are in clinical use for non-cancerous indications. Hence, further investigations of ROCK inhibitors in combination with other drugs should be performed. Since neuroblastomas can be regarded as a disease caused by obstructed cell differentiation during embryonal development, agents that release this differentiation block are attractive as potential new therapies for high-risk patients.

## Targeted Therapy for Neuroblastoma—ALK, a Success Story

ALK, the second most common driver in neuroblastoma after MYCN, is a receptor tyrosine kinase which in the neuroblastoma context first attracted attention in conjunction with the rare hereditary neuroblastomas [15]. The oncogenic mechanism in neuroblastoma operates by activation of RAS-MAPK-ERK and other downstream pathways, and can be initiated by activating mutations of *ALK*, *ALK* amplifications, or, possibly, changes to the ALK ligands ALKAL1 and ALKAL2 [82–84]. The *ALK* gene is situated in proximity to *MYCN* and *ALKAL2* on chromosome 2p and it has been argued that co-amplification of these three explains the inferior outcome in neuroblastoma patients with 2p gain [85].

Among targeted therapies proposed for neuroblastoma treatment, ALK inhibitors have come furthest in clinical application. The first clinical trial of the lead substance crizotinib (NCT00939770) started shortly after ALK was discovered as an oncogenic driver in neuroblastoma [86]. While there were some responses in a subset of neuroblastomas, most patients did not respond. Fueled by advances in the field of ALK-positive non-small cell lung cancer (NSCLC), where ALK fusions constitute the predominant driving oncogenic event and where the initial response is frequently followed by the development of acquired resistance, second- and third-generation ALK inhibitors soon became available, including ceritinib, brigatinib, entrectinib, and lorlatinib. Some of these agents also inhibit other tyrosine kinases that may be of importance in neuroblastoma, e.g., ROS1 or TRK. Eventually, it became evident that different ALK hotspot mutations in neuroblastoma confer differential sensitivity toward different ALK inhibitors [87–89]. In selected cases, individualized preclinical evaluation of a newly described ALK or ALK ligand mutation has proven beneficial [84, 90]. Secondary resistance toward ALK inhibitors is a frequent observation in NSCLC patients and is also seen in neuroblastoma patients, and sometimes the switch to an alternative ALK inhibitor becomes necessary [91]. In general, ALK inhibitors cause low toxicity, allowing their use even under circumstances when chemotherapy cannot be tolerated [90]. A number of responders have remained on ALK inhibitor treatment well beyond 1 year [84, 86, 88, 90, 92].

Clinical experience with ALK inhibition in neuroblastoma has hitherto mainly been collected in patients with refractory or relapsed tumors. For neuroblastoma with *ALK* mutations, the TITAN study (transatlantic integration targeting ALK in neuroblastoma) will investigate the frontline addition of lorlatinib, an ALK inhibitor with activity against a wider range of ALK mutations and penetrance to CNS [93–95]. In this context, liquid biopsies for ALK mutations might prove useful to monitor lorlatinib treatment response [96].

## Conclusions

The rapid advances in Omics techniques and analysis of “big data” have greatly increased our knowledge of the molecular landscape of neuroblastoma. National and international molecular profiling initiatives have provided bioinformatic pipelines to guide treatment options of individual patients with the potential to be implemented as personalized medicines [97].

The new insights concerning the molecular heterogeneity, plasticity, and the involvement of non-tumorigenic cells within the neuroblastoma tumor microenvironment have advanced our understanding of the initiation, progression, and metastatic spread of neuroblastoma. However, these new discoveries also call for new models that more accurately mimic the disease in order to design accurate functional studies and to monitor the effects of novel drug regimens. Patient-derived xenograft models and the establishment of organoids that more accurately resemble the patient tumor may better predict the effects of specific drugs which could then possibly be directly transferred to clinical use if the toxicity profile is acceptable. The advancement in the molecular biology and pathogenesis of neuroblastoma has opened up the possibility to apply more precise treatment to children with this disease. Hopefully, this could lead to the cure of patients whom we currently are unable to cure.
